# Role of Growth Differentiation Factor 15 in Lung Disease and Senescence: Potential Role Across the Lifespan

**DOI:** 10.3389/fmed.2020.594137

**Published:** 2020-12-03

**Authors:** Faeq Al-Mudares, Samuel Reddick, Jenny Ren, Akshaya Venkatesh, Candi Zhao, Krithika Lingappan

**Affiliations:** ^1^Department of Pediatrics, Baylor College of Medicine, Houston, TX, United States; ^2^Rice University, Houston, TX, United States; ^3^Baylor College of Medicine, Houston, TX, United States

**Keywords:** growth differentiation factor 15, senescence, chronic obstructive pulmonary disease, lung fibrosis, bronchopulmonary dysplasia, pulmonary hypertension

## Abstract

Growth Differentiation Factor 15 (GDF15) is a divergent member of transforming growth factor-beta (TGF-β) superfamily and is ubiquitously expressed, under normal physiological conditions. GDF15 expression increases during many pathological states and serves a marker of cellular stress. GDF15 has multiple and even paradoxical roles within a pathological condition, as its effects can be dose- and time-dependent and vary based on the targeted tissues and downstream pathways. GDF15 has emerged as one of the most recognized proteins as part of the senescence associated secretory phenotype. Cellular senescence plays a major role in many lung diseases across the life-span from bronchopulmonary dysplasia in the premature neonate to COPD and idiopathic pulmonary fibrosis in aged adults. GDF15 levels have been reported to be as a useful biomarker in chronic obstructive pulmonary disease, lung fibrosis and pulmonary arterial hypertension and predict disease severity, decline in lung function and mortality. Glial-cell-line-derived neurotrophic factor family receptor alpha-like (GFRAL) in the brain stem has been identified as the only validated GDF15 receptor and mediates GDF15-mediated anorexia and wasting. The mechanisms and pathways by which GDF15 exerts its pulmonary effects are being elucidated. GDF15 may also have an impact on the lung based on the changes in circulating levels or through the central action of GDF15 activating peripheral metabolic changes. This review focuses on the role of GDF15 in different lung diseases across the lifespan and its role in cellular senescence.

## Introduction

Growth Differentiation Factor 15 (GDF15) is a stress-responsive cytokine that mediates anorexia and cachexia in many chronic diseases and cancer. GDF15 levels can predict all-cause mortality in a multitude of diseases ranging from heart failure to cancer (1–5). It is also emerging as a cell-autonomous modulator of cellular senescence, as a member of the senescence associated secretory phenotype (SASP) protein repertoire. We attempted to review the role of GDF15 in lung diseases in which cellular senescence plays a key role. GDF15 is a divergent member of transforming growth factor-beta (TGF-β) superfamily, which was first discovered by Bootcov et al. (6). The mature proteins of TGF-β superfamily share a cysteine knot that contains a highly conserved seven-cysteine domain. The degree of similarity within the amino acid sequence of this domain is used to categorize the superfamily members into their specific family groups. The GDF15 protein molecule does not show a high enough homology to any existing family group. Therefore, it is identified as a divergent member within the TGF-β superfamily (6, 7). GDF15 has very high expression levels in organs including the placenta (8), the prostate (7), and the liver (9). High expression levels have been reported in epithelial cells and macrophages (10).

The circulatory levels of GDF15 in adults can range between 0.1 and 1.2 ng/ml (11). In physiological states, GDF15 is weakly expressed except during pregnancy, during which, expression continues to increase throughout gestation (12). GDF15 plays an important role at the maternal-fetal interface to facilitate placentation and to maintain pregnancy possibly via its immunosuppressive effect (13). GDF15 expression increases during pathological states including inflammation, aging, smoking, cancer (14), oxidative stress (15), and hypoxia (16). It may have multiple and even opposing roles within the same pathological state, as its effects can be dose- and time-dependent, and vary based on the targeted tissues and downstream activated pathways. Other aliases for GDF15 include macrophage inhibitory cytokine-1 (MIC-1) (6), non-steroidal anti-inflammatory drug activated gene-1 (NAG-1) (17), placental transforming growth factor-beta (PTGFβ) (18), and prostate-derived factor (PDF) (7).

## Regulation of Expression and Signaling

GDF15 expression in the macrophages is induced significantly by interleukin-1beta (IL-1β) and tumor necrosis factor-alpha (TNF-α). GDF15 expression inhibits TNF-α production in macrophages revealing its autocrine regulatory effect on the macrophages, hence its other name MIC-1 (6).

Many transcription factors have been identified to increase GDF15 expression. The GDF15 promoter has two p53 binding sites, p53 induces GDF15 which in turn modulates p53-mediated growth suppression in tumor cells in a paracrine manner (44). Early growth response gene-1 (EGR-1) and Sp1 share the same DNA binding site on the GDF15 promoter. Additionally, peroxisome proliferator-activated receptor gamma (PPAR-γ) and activating transcription factor-3 (ATF-3) act as positive regulators that can induce GDF15 expression (14, 45). Interestingly, anoxia induces GDF15 expression independent of p53 (16). NSAIDs induce GDF15 expression in a cyclooxygenase- and p53-independent manner (45).

The glial-cell-line-derived neurotrophic factor family receptor alpha-like (GFRAL) was recently identified as a receptor with a high affinity to GDF15. GFRAL is only known to be expressed in the nerve cells of the brain stem specifically in the area postrema and the nucleus tractus solitarius (30, 46, 47). RET is a known coreceptor for different GFRA family members, and its presence is necessary for GDF15 signaling. Binding of GDF15 to GFRAL-RET complex activates downstream signaling involving ERK, AKT and PLC-γ (48). GDF15 may mediate its anorexia effects by acting on these receptors. However, the recent use of monoclonal antibodies inhibiting GFRAL signaling reversed the GDF15-induced cachexia even under calorie-restricted conditions, revealing that GDF15 may lead to decreased adipose and muscle mass and function independently of anorexia (31). This was mediated through its lipolytic effect in the adipose tissue via the sympathetic axis. It is currently unclear as to which receptors mediate the action of GDF15 in other tissues and organs including the lung. Zhang et al. concluded neither GFRAL nor it's co-receptor RET was expressed in the lung (both by immunohistochemistry and query of the sc-RNASeq data). TGFBR1 and TGFBR2 on the other hand are expressed in the lung. Whether GDF15 binds to these receptors in the lung is not known (41). GDF15 mediated effects on other organs have been associated with activation of other pathways. The anti-hypertrophic effect on the cardiac muscle mediated by GDF15 has been reported to be mediated through the activation of SMAD2/3 pathway (49). Through its central actions in the brain similar to a hormone, GDF15 may also exert changes in metabolism in peripheral tissues. This has been shown in a sepsis model, where GDF15 increased triglyceride metabolism by increasing the sympathetic outflow to the liver and improved survival (50).

## Role of GDF15 in Chronic Lung Disease: Neonatal and Pediatric

Early life exposure to adverse stimuli can predispose the lung to premature senescence and accelerate deterioration of lung function with a second hit such as smoking or viral infections (51). Many molecular pathways that are associated with aging are also altered in the neonatal BPD lung (51, 52). Hyperoxia is known to induce a senescent phenotype in many cell types including airway smooth muscle cells and fibroblasts (53–55).

During fetal lung development, GDF15 expression seemed to be involved in the promotion of proliferation and differentiation and was androgen-responsive (19). The circulating levels of GDF15 in full-term newborns are 10-fold higher than adult levels, that soon after birth trend down to reach adult levels between 4 and 12 months (20). The circulatory levels of GDF15 in preterm infants across different gestational age windows have not been studied.

Bronchopulmonary dysplasia (BPD) is one of the most common causes of long-term morbidity among surviving preterm infants (56). BPD is characterized by arrest in lung development leading to significant aberrant vascular development and alveolar septation (57). Hyperoxia is one of the causative factors for the development of BPD. GDF15 is a part of the stress response pathway and is activated in response to hyperoxia exposure. Our published data showed that hyperoxia significantly induced GDF15 expression in pulmonary epithelial and endothelial cells. Additionally, GDF15 loss decreased cell viability and increased oxidative stress in hyperoxic conditions (22, 23). GDF15 was one of the top upregulated genes in the lung in an independent study in hyperoxia-exposed neonatal mice (21). The role of GDF15 in neonatal and pediatric lung disease needs further research toward elucidating its role in alveolarization and pathogenesis of chronic lung disease related to prematurity and other pediatric conditions.

## Role of GDF15 in Chronic Obstructive Pulmonary Disease (COPD)

COPD is one of the leading causes of morbidity and mortality worldwide, and it is expected to pose even a higher health problem in the future (58). COPD is a heterogeneous disease that is commonly associated with recurrent exacerbations, cachexia, and high mortality (59). Compared to healthy control subjects, circulating GDF15 concentrations are 2.1-fold higher in stable COPD patients (60). GDF15 is associated with COPD severity, exacerbation, and prognosis in several independent study cohorts. GDF15 levels independently contributed to the risk of subclinical coronary atherosclerosis (24) and to higher mortality, exacerbation rates, and decline in lung function (4). While the regulatory pathways of GDF15 production are not fully elucidated, its expression is induced by key features of COPD, including hypoxia, intracellular oxidative stress, and increased inflammatory cytokines (6, 16, 61).

Cigarette smoking is the leading cause and the single most significant risk factor for developing COPD (62). In human small airway epithelial cells, cigarette smoke (CS) extract induces GDF15 expression (25). CS increases GDF15 expression in humans with and without COPD and in murine models for COPD (26). Significantly, deletion of the GDF15 gene in mice led to attenuation of CS related pulmonary inflammation (26). GDF15 also plays an important role in airway mucosal immunity as it promotes mucin production via activation of phosphoinositide 3-kinase (PI3K) in the ciliated epithelial cells (25, 28, 63). CS-induction of GDF15 also promotes cellular senescence through the activin receptor-like kinase 1/Smad1 pathway and increases expression of cellular senescence markers including p21, p16, and high-mobility group box 1 (HMGB1) in airway epithelial cells (27).

Low body mass index (BMI) is a risk factor for exaggerated decline in the lung function, and is an independent predictor of the overall mortality in COPD (64), and is a bad prognostic indicator in the BODE index; which is used to predict the mortality risk among COPD patients (65). Elevated levels of GDF15 are associated with low muscle mass and strength, in aging patients (66, 67). In COPD patients, it was associated with a sedentary lifestyle and cognitive risk (68). High GDF15 levels have also been associated with higher risk of ICU-acquired muscle weakness (69). The recent discovery of GFRAL in the hindbrain may explain the role GDF15 in the loss of muscle mass noted in patients with COPD, probably mediated though cachexia, similar to cancer patients. In addition, GDF15 promotes muscle wasting *in vivo* and lipolysis in adipose tissue *in vitro*, highlighting other possible mechanisms of GDF15-induced muscle wasting and decrease in BMI (32, 33).

Patients with COPD are susceptible to acute exacerbations of their lung disease. In transgenic mice expressing human GDF15, lung inflammation was increased secondary to human rhinovirus infection by GDF15. Since CS exposure increases GDF15 expression at baseline, this could predispose patients with COPD to respiratory viruses and lead to acute exacerbation of their lung disease (29). GDF15 levels are positively correlated with a poor outcome in patients with acute respiratory distress syndrome (70, 71). However, in other experimental models of acute lung injury, GDF15 has been shown to be protective, which suggests that it may be part of the body's defense response. Herter et al. showed that in a model of ventilator induced lung injury that GDF15 attenuated lung injury by decreasing the formation of platelet-neutrophil aggregates (72). In an lipopolysaccharide (LPS)-induced acute lung injury model, the protective effect of GDF15 was neutralized after suppression of SIRT1 (73).

## Role of GDF15 in Pulmonary Hypertension (PH)

GDF15 levels are associated with PH risk and progression. Elevated GDF15 levels were associated with increased right atrial and pulmonary capillary wedge pressure and were an independent predictor of adverse outcomes (34), and with a higher rate of mortality, transplantation, and heart failure (35). In a study evaluating novel cardiovascular biomarkers that reflect different pathobiological pathways, GDF15 levels were significantly elevated in PH patients and were primarily associated with left-sided heart disease or post-capillary PH (74). Additionally, the decline in GDF15 levels were correlated with hemodynamic improvement following balloon pulmonary angioplasty in patients with chronic thromboembolic pulmonary hypertension (36). In pediatric patients with PH associated with congenital heart disease plasma GDF15 were considerably elevated and had a similar diagnostic power as NT-proBNP (37).

Similar to cancer and COPD, in PH models, GDF15 contributed to skeletal muscle atrophy through increased phosphorylation of TGFβ-activated kinase 1 (TAK1) (38). GDF15 is upregulated due to shear stress and hypoxia in areas of active vascular remodeling in human microvascular endothelial cells in PH, especially in plexiform lesions (75). In hypoxic human umbilical vein endothelial cells (HUVECs) (39), GDF15 inhibited p53 signaling, stabilized HIF-1 alpha and promoted angiogenesis. GDF15 also prevented high glucose-induced endothelial cell apoptosis in HUVECs (40). In summary, GDF15 could play a functional role in the pathogenesis of vascular lesions in PH and may be associated with risk, progression, and mortality.

## Role of GDF15 in Lung Fibrosis

Idiopathic pulmonary fibrosis is one of the major fibrotic lung diseases with major morbidities and high mortality among adults and is associated with lung epithelial cell dysfunction. In an animal model of type-2 alveolar epithelial cell specific telomere dysfunction, GDF15 was the most significantly upregulated protein in senescent type II alveolar epithelial cells. GDF15 expression was also increased in human IPF lungs (41). In a landmark study, that sought to identify the associations between aging related biomarkers and interstitial lung abnormalities as a prelude to the development of IPF. Subjects from two large study cohorts: The Framingham Heart Study (FHS) and Genetic Epidemiology of COPD Study (COPDgene) were included. Significantly, high levels of GDF15 was associated with higher odds of interstitial lung abnormalities in both the cohorts. Additionally, the study showed that elevated GDF15 levels in the blood may precede pulmonary fibrosis development, and may mediate the association between aging and interstitial lung abnormalities (42).

Lung epithelial cells were identified as the primary source of GDF15 production in human lungs through single-cell sequencing (41). Similar results were reported in a bleomycin model of pulmonary fibrosis, GDF15 expression was increased in the lung, bronchoalveolar lavage fluid and plasma and was associated with markers of cellular senescence in alveolar epithelial cells (76). Interestingly, Lambrecht et al. showed no differences in lung fibrosis development between GDF15 deficient mice compared to the wild type when exposed to bleomycin (77). However, GDF15 levels were increased in the bleomycin-exposed lungs and there was decreased expression of IL-6 and CCL2 in the lung fibroblasts from the GDF15 deficient mice, upon exposure to bleomycin. GDF15 may have profibrotic properties by activating fibroblasts and M2 macrophages (76), or prevent the overactivation of fibroblasts during lung remodeling via inactivation of the TGF-Smad pathway (43). GDF15 may activate different distal signaling pathways in different cell types, potentially explaining why GDF15 appears to have both protective and deleterious effects. Additionally, GDF15 may act as a partial agonist on the TGF-β receptor, explaining its dose-dependent effect. Significantly, circulating concentrations of GDF15 are elevated and correlate with severity of disease in IPF patients (41).

GDF15 levels were also increased in patients with systemic sclerosis and correlated with clinical symptoms of lung fibrosis and deterioration in lung function (78). Determining the nature of GDF15's role in pulmonary fibrosis will be crucial to elucidating how GDF15 may be used alone or in combination as a diagnostic, prognostic biomarker, or both. From there, investigators can determine the utility of GDF15 may be a potential therapeutic target.

## Role of GDF15 in Senescence

GDF15 is emerging as a major cell autonomous mediator of cellular senescence (79, 80). Cellular senescence is marked by the activation of senescence-associated secretory-phenotype (SASP) (41, 81), which corresponds with the secretion of a repertoire of proteins that are thought to promote inflammation and cellular dysfunction. Tanaka et al. characterized the plasma proteomic signature in healthy aging humans and found that GDF15 had the strongest positive association with age (82). GDF15 was found to be one of the proteins in the SASP repertoire in many other studies, indicating the possibility for GDF15 to modulate and/or predict senescence (41, 83–88). Over expression of human GDF15 in female mice extended lifespan in a study by Wang et al. (89). However, GDF15 is also known as a stress responsive cytokine so the clear delineation between the role of GDF15 in mediating cellular senescence and as a biomarker for cellular stress needs to be explored. Lockhart et al. suggested that the senescent cells lead to the development of frailty via increasing levels of GDF15 that act centrally, resulting in appetite suppression (90).

GDF15 was secreted by senescent endothelial cells along with osteopontin and IL-8 (85) and activated the ROS-mediated p16 pathway (80). Similar results were shown in senescent adult blood endothelial colony forming cells which secreted increased GDF15, which in turn had a beneficial paracrine effect on non-senescent endothelial cells (91). GDF15 bound to activin receptor-like kinase 1 receptor and activated the Smad1 pathway to cause senescence in cigarette-smoke exposed airway epithelial cells (27). A recent review by Conte et al. discussed the role of GDF15 as a mitokine; a soluble molecule secreted during mitochondrial stress that may have paracrine effects, and play a role in modulation age-related inflammatory processes and immunosenescence, as part of an adaptive mechanism to aging (92). GDF15 contributes to the TReg-mediated suppression of conventional T-cell activation and inflammatory cytokine production in senescence (93). Though GDF15 expression seems to be increased in different type of senescent cells, whether it has a protective or adverse paracrine effects on adjoining cells or even effects on distant organs through circulating levels needs to be discerned.

## Conclusion

**Figure 1:**
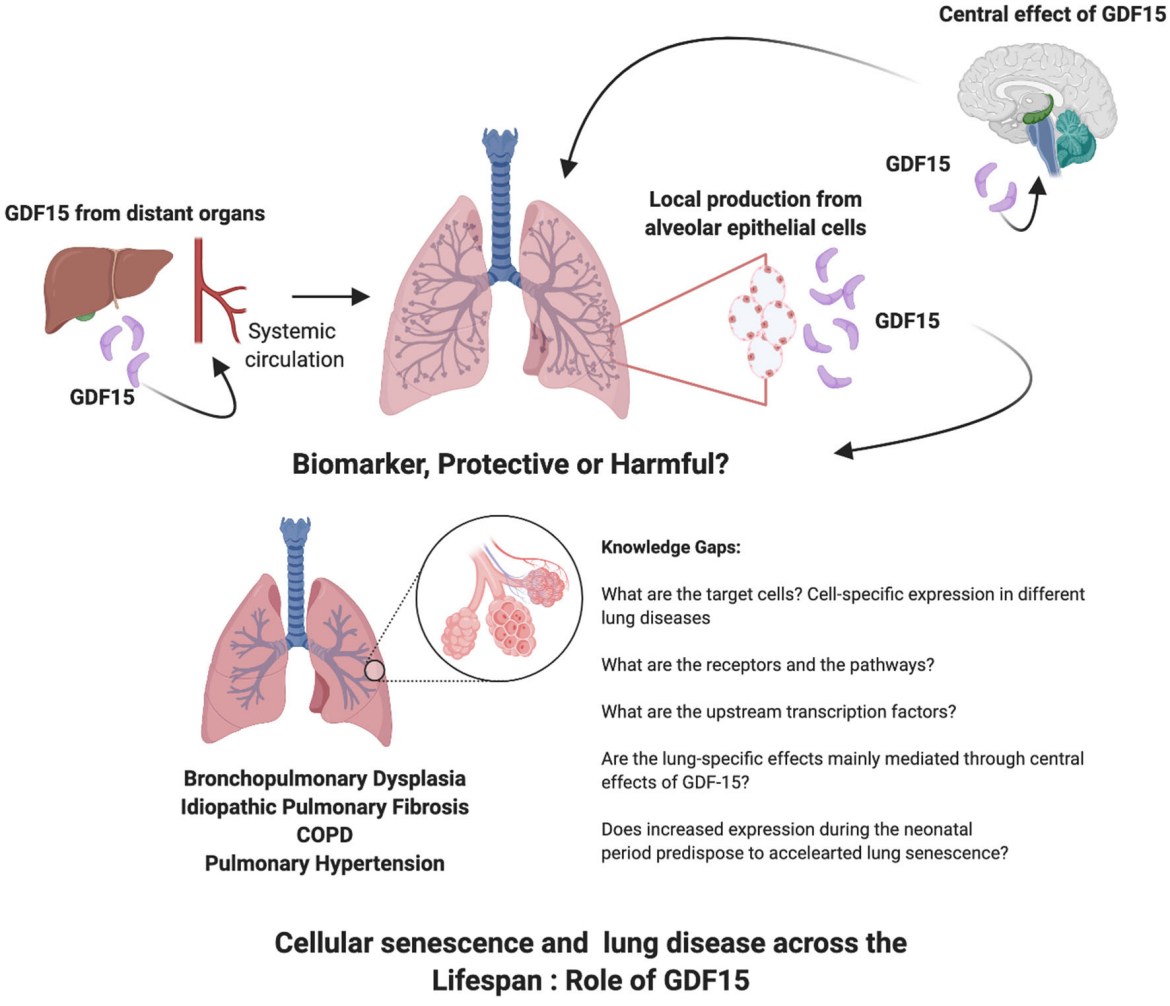
Cellular senescence and lung disease across the lifespan: role of GDF15.

GDF15 contributes to the pathophysiology of many lung diseases in which senescence plays a role as well (Table 1). It is emerging as one of the proteins that is strongly associated with aging and is a part of the cellular response to activation of senescence related pathways (Figure 1). What is unknown are the receptors and pathways through which GDF15 may exert direct effects on the various lung cellular sub-populations. Targeted deletion or overexpression of GDF15 in the lung cells will be necessary to answer these questions. The lung epithelial cells are the most probable source but other cells may also secrete GDF15 under different pathological stressors. Also, since many other organs such as the liver produce GDF15 the effects on lung under pathological conditions may be secondary to circulating levels of GDF15 or mediated through a central action of GDF15. Based on the current state of literature about GDF15, its role in cellular senescence and in lung diseases across the lifespan, the following gaps on the role of GDF15 in lung diseases are identified: Do recruited inflammatory cells increase GDF15 levels in the lung? Are epigenetic mechanisms involved in the GDF15 expression and reprogramming of cellular senescence or acceleration of premature senescence especially when organ injury happens early in life, in the neonatal or pediatric period? Are the effects on peripheral organs such as the lung mainly mediated through the central effects of GDF15, which then cause changes in the target organ metabolism through sympathetic pathways? Is GDF15 expression an adaptive, protective or harmful response in a specific lung disease? What are the transcription factors upstream modulating GDF15 expression in the spectrum of lung diseases? Would GDF15 be most useful as a biomarker or a therapeutic target? The interest in GDF15 as a senescence associated protein is growing, and so is the role of senescence in lung diseases, the intersection between the two reveals many areas of research which will elucidate the role of GDF15.

**Table 1:** List of GDF15 functions and mechanisms on different lung diseases.

**Age/Condition**	**Role of GDF15**	**References**
Pregnancy and neonatal period/Bronchopulmonary dysplasia (BPD)	Promotes proliferation and differentiation in fetal lung development	(19)
	Maternal serum levels increase throughout during pregnancy	(12)
	High serum levels in term neonates that decline postnatally	(20)
	Upregulated in neonatal mice exposed to hyperoxia *in vivo*	(21)
	Upregulated in pulmonary epithelial and endothelial cells exposed to hyperoxia	(22)
	GDF15 loss leads to decreased cell viability and increased oxidative stress	(23)
Chronic Obstructive Pulmonary Disease (COPD)	Higher serum levels are associated with increased morbidity and mortality	(4, 24)
	Mediates smoking-induced inflammation and cellular senescence	(25–27)
	Promotes mucin production in ciliated epithelial cells	(28)
	Exacerbates lung inflammation secondary to infection	(29)
	Contributes to cachexia: GFRAL mediated signaling, induces lipolysis and promotes muscle wasting	(30–33)
Pulmonary Hypertension (PH)	Associated with prognosis and response to therapy	(34–36)
	Levels increased in pediatric PH related to congenital heart disease	(37)
	Associated with increase in right atrial and pulmonary capillary wedge pressure	(34)
	Induces muscle atrophy that is reversed by TAK1 inhibitor	(38)
	Promotes angiogenesis and hinders endothelial cell apoptosis	(39, 40)
Lung Fibrosis	Associated with disease severity	(41)
	Associated with higher odds of interstitial lung abnormality	(42)
	Activates fibroblasts and M2 macrophages	(40)
	Prevents the activation of fibroblasts during lung remodeling	(43)

## Author Contributions

FA-M contributed significantly to conceptualizing, drafting, and revising the manuscript. SR contributed by drafting and revising the COPD and pulmonary hypertension sections. JR contributed by drafting and revising the lung fibrosis section. AV contributed by drafting and revising the senescence section. CZ contributed by drafting and revising the neonatal and pediatric section. KL contributed significantly to conceptualizing, reviewing, and revising the manuscript. All authors approved the final manuscripts as submitted and agree to be accountable for all aspects of the work.

## Conflict of Interest

The authors declare that the research was conducted in the absence of any commercial or financial relationships that could be construed as a potential conflict of interest.
